# Advanced Age Is Associated with Iron Dyshomeostasis and Mitochondrial DNA Damage in Human Skeletal Muscle

**DOI:** 10.3390/cells8121525

**Published:** 2019-11-27

**Authors:** Anna Picca, Robert T. Mankowski, George Kamenov, Stephen D. Anton, Todd M. Manini, Thomas W. Buford, Sunil K. Saini, Riccardo Calvani, Francesco Landi, Roberto Bernabei, Emanuele Marzetti, Christiaan Leeuwenburgh

**Affiliations:** 1 Institute of Internal Medicine and Geriatrics, Università Cattolica del Sacro Cuore, 00168 Rome, Italy; anna.picca1@gmail.com (A.P.); riccardo.calvani@gmail.com (R.C.); francesco.landi@unicatt.it (F.L.); roberto.bernabei@unicatt.it (R.B.); 2 Fondazione Policlinico Universitario “Agostino Gemelli” IRCCS, 00168 Rome, Italy; 3 Department of Aging and Geriatric Research, Institute on Aging, University of Florida, Gainesville, FL 32611, USA; r.mankowski@ufl.edu (R.T.M.); santon@ufl.edu (S.D.A.); tmanini@ufl.edu (T.M.M.); sunil.saini@ufl.edu (S.K.S.); cleeuwen@ufl.edu (C.L.); 4 Department of Geological Sciences, University of Florida, Gainesville, FL 32605, USA; kamenov@ufl.edu; 5 Department of Medicine, University of Alabama at Birmingham, Birmingham, AL 35205, USA; twbuford@uabmc.edu

**Keywords:** iron overload, hepcidin, transferrin, ferritin, ZIP, inflammation, mtDNA, mitochondrial dysfunction, muscle aging, physical performance

## Abstract

Whether disruption of iron metabolism is implicated in human muscle aging is presently unclear. We explored the relationship among iron metabolism, muscle mitochondrial homeostasis, inflammation, and physical function in older adults and young controls. Eleven young and 23 older men and women were included. Older adults were classified into high–functioning (HF) and low–functioning (LF) groups according to their Short Physical Performance Battery score. Vastus lateralis muscle biopsies were assayed for total iron content, expression of 8-oxoguanine and DNA glycosylase (OGG1), 3-nitrotyrosine (3-NT) levels, and mitochondrial DNA (mtDNA) content and damage. Circulating ferritin and hepcidin levels were also quantified. Muscle iron levels were greater in the old group. Protein expression of transferrin receptor 1, Zrt-Irt-like protein (ZIP) 8, and ZIP14 were lower in old participants. Circulating levels of ferritin, hepcidin, interleukin 6 (IL6), and C-reactive protein were higher in the old group. Old participants showed lower mtDNA content and greater mtDNA damage. OGG1 protein expression declined with age, whereas 3-NT levels were greater in old participants. Finally, a negative correlation was determined between ZIP14 expression and circulating IL6 levels in LF older adults. None of assayed parameters differed between HF and LF participants. Our findings suggest that muscle iron homeostasis is altered in old age, which might contribute to loss of mtDNA stability. Muscle iron metabolism may therefore represent a target for interventions against muscle aging.

## 1. Introduction

Iron is the most abundant transition metal in living organisms and is involved in multiple biochemical processes including oxygen binding and transport, energy production, regulation of cell growth and differentiation, and a variety of enzyme reactions. Most body iron is incorporated into haem proteins (e.g., haemoglobin, myoglobin, cytochromes, and haem thiolates). Non-haem iron serves instead as an enzyme cofactor (i.e., atomic iron) or iron reserve (e.g., bound to cytosolic ferritin and haemosiderin) and is integral to electron transport chain complexes (i.e., iron-sulphur clusters) and transferrin (Tf) [1,2]. Approximately 5% of cellular iron exists as chelatable non-haem iron, referred to as labile iron pool. This iron fraction consists of both ferrous (Fe^2+^) and ferric (Fe^3+^) ions associated with a variety of small molecules, including organic anions, polypeptides, and phospholipids. Fe^2+^ ions can participate in Fenton reactions thereby producing highly destructive radicals, which are thought to be major contributors to the generation of protein and DNA oxidative adducts [3,4,5]. Hence, a tight coordination encompassing iron absorption, uptake, efflux, and sequestration is crucial to preserve cell homeostasis.

Circulating iron is bound and transported by Tf. However, in the setting of iron overload, the iron-binding capacity of plasma Tf can be exceeded and accumulation of non-Tf-bound iron (NTBI) occurs [6]. As such, NTBI needs to be adequately disposed. Fourteen divalent metal transporters belonging to the Zrt-Irt-like protein (ZIP) family, named ZIP1 to ZIP 14, have been identified [7]. Of them, ZIP8 and ZIP14 have similar amino acid sequences [8] and contribute to the import of several divalent ions, including iron [9]. In particular, Zip14 mediates, at least in part, NTBI uptake by hepatocytes in the context of iron overload [10].

Iron metabolism is modulated by the defensin-like hormone hepcidin [11] via binding and subsequently degrading of the iron exporter ferroportin at the level of key iron sources [i.e., duodenal enterocytes (absorption of dietary iron), splenic and hepatic macrophages (recycling iron from erythrophagocytosis), and hepatocytes (iron stores)] [12]. In particular, circulating iron concentrations decrease as a consequence of intestinal absorption and release of iron from recycling macrophages [11].

Skeletal muscle is a major reservoir of body iron, which is comprised by 60% of non-haem fraction [13]. Studies have shown that non-haem iron accumulates in muscle during ageing possibly causing oxidative damage to biomolecules and organelles, including mitochondria [14,15,16,17,18]. As such, iron dyshomeostasis is advocated as a mechanism involved in the pathogenesis of sarcopaenia of ageing and disuse-induced muscle atrophy [19].

Along with iron imbalance and mitochondrial dysfunction, chronic inflammation is a hallmark of ageing and a factor involved in functional decline [20,21]. A link between mitochondrial damage and chronic low-grade inflammation has recently been hypothesised [21,22]. However, little is known about the relationship among iron dyshomeostasis, inflamm-ageing, mitochondrial dysfunction, and physical performance in older adults. To provide an initial appraisal of the subject, the present study was undertaken to assess total iron content, the expression of selected iron transporters, and indexes of mitochondrial damage in muscle biopsies obtained from healthy young adults and older people with varying levels of physical performance. The relationship between muscle iron content and systemic inflammation was also explored.

## 2. Materials and Methods

### 2.1. Participants

Participants were community-dwelling men and women aged 70 years or older. Healthy young adults between the ages of 18 and 35 years were recruited as controls. Participant recruitment was coordinated by the Recruitment Core of the University of Florida Claude D. Pepper Older Americans Independence Center, as detailed elsewhere [23,24,25].

A set of eligibility criteria was chosen to minimise the possible confounding effect of co-morbid conditions, medications, or lifestyle habits on the relationship among physical performance, iron metabolism, and indexes of muscle mitochondrial damage [26]. Briefly, candidates were not included if presenting with any of the following characteristics: smoking in prior 12 months; engagement in regular physical exercise; history of drug or alcohol abuse; active treatment for cancer or cancer in the past three years; heart failure New York Heart Association class III–IV; stroke with upper and/or lower extremity involvement; Parkinson’s disease or other neurological disorders likely to interfere with physical function; major psychiatric illnesses; peripheral vascular disease Lériche–Fontaine stage 3–4; history of life-threatening cardiac arrhythmias; cognitive impairment (i.e., Mini Mental State Examination score ≤ 21); renal disease requiring dialysis; lung disease requiring steroids; chronic viral diseases (e.g., hepatitis B and C, HIV); lower extremity amputation; severe knee or hip osteoarthritis limiting mobility; diabetes with visual, vascular or neuropathic complications; inflammatory diseases (e.g., rheumatoid arthritis, vasculitis, autoimmune disorders, inflammatory bowel disease); taking growth hormone, oestrogen replacement, testosterone, anticoagulants, steroids, or non–steroidal anti–inflammatory drugs on a regular basis; severe obesity [i.e., body mass index (BMI) ≥35]; underweight (i.e., BMI ≤18.5); active weight loss >5 kg in prior three months; life–threatening illnesses with an estimated life expectancy <1 year. Candidates on statin treatment were asked to refrain from drug administration one month prior to blood drawn upon their general practitioner’s approval.

Old enrolees were categorised as high–functioning (HF) and low–functioning (LF) based on their Short Physical Performance Battery (SPPB) summary score [27]. Specifically, participants with a SPPB score ≥11 were classified as HF, while those who scored ≤7 were categorised as LF. These cut-offs were selected based on their ability to predict several relevant health outcomes in older adults (e.g., functional limitations, institutionalisation, mortality) [27,28,29,30,31]. Individuals scoring 8–10 on the SPPB were excluded to allow greater discrimination in physical function and possibly biochemical parameters between groups.

Prior to enrolment in the study, all participants provided written informed consent. The study protocol was approved by the University of Florida’s Institutional Review Board (IRB201300790).

### 2.2. Blood Collection and Processing

Blood samples were obtained in the morning by venipuncture of the median cubital vein after overnight fasting, using commercial ethylenediaminetetraacetic acid (EDTA) collection tubes (BD Medical, Franklin Lakes, NJ, USA). Samples were immediately centrifuged at 1000× *g* for 10 min at 4 °C, aliquots were prepared, and stored at −80 °C until analysis.

### 2.3. Collection of Muscle Biopsies

Muscle samples were obtained from the vastus lateralis of the dominant lower extremity by percutaneous needle biopsy, under local anaesthesia, as previously described [25]. Muscle specimens were cleaned of any visible blood and fat, snap-frozen in liquid nitrogen, and subsequently stored at −80 °C until analysis.

### 2.4. Measurement of Circulating Iron Transporters and Inflammatory Biomarkers

Plasma levels of the iron transporter ferritin and the iron regulator hepcidin as well as those of C-reactive protein (CRP) and interleukin (IL) 6 were measured using enzyme-linked immunosorbent assays (ferritin: Human ELISA Kit, Thermo Scientific (Waltham, MA, USA); hepcidin: Intrinsic Hepcidin IDx™ ELISA Kit, Intrinsic LifeSciences (La Jolla, CA, USA); CRP: Human C-Reactive Protein/CRP Quantikine ELISA Kit, R&D Systems (Minneapolis, MN, USA); IL6: Human IL-6 Quantikine HS ELISA Kit, R&D Systems). Plate processing and data collection were carried out according to the manufacturer’s instructions. Absorbance was read on a Synergy HT Multi-Detection microplate reader (BioTek, Winooski, VT, USA). Concentrations of ferritin, hepcidin, and CRP are shown as ng/mL, whilst IL6 levels are reported in pg/mL.

### 2.5. Inductively Coupled Plasma-Mass Spectrometry (ICP-MS) Determination of Total Iron in Muscle Biopsies

Total iron content in muscle samples was determined by ICP-MS as described previously with modifications [32]. Briefly, 15–30 mg of vastus lateralis muscle samples were digested in 1 mL concentrated nitric acid (HNO_3_ Optima-grade) in capped Teflon (Savillex Corporation, Eden Prairie, MN, USA) vials for 24 h. Afterwards, 1 mL of 30% hydrogen peroxide (H_2_O_2_ Optima-grade) was added to each vial and placed opened on a hot plate (100 °C) to let the mixture evaporate. Subsequently, 1 mL of HNO_3_ and 1 mL of H_2_O_2_ were added to the dry residue and incubated on the hot plate (100 °C) overnight to digest any remaining organic material. After this second digestion, samples were evaporated to dryness, followed by addition of 0.8 N HNO_3_ spiked with 8 parts per billion (ppb) rhenium (Re) and rhodium (Rh). Vials were then incubated at 100 °C overnight to ensure complete dissolution. A fraction of the sample solution was removed and further diluted with 0.8 N HNO_3_ spiked with 8 ppb Re and Rh to obtain a final dilution of approximately 300×. The exact final dilution for elemental analyses was achieved according to the weight of each sample. Trace element analysis was conducted on a Thermo Finnigan Element2™ high–resolution ICP-MS (Thermo Fisher Scientific, San Jose, CA, USA) in medium resolution using Re and Rh as internal standards. In order to avoid analytical biases, all samples were run in the same day and in the same sequence. Results were quantified by external calibration using a combination of gravimetrically prepared ICP-MS standards obtained from QCD Analysts (www.qcdanalysts.com). Iron concentrations are reported in parts per million (ppm), with an analytical error < ±5%.

### 2.6. Western Immunoblotting

Protein content of Tf receptor 1 (TFR1), ZIP8, ZIP14, and 8-oxoguanine DNA glycosylase (OGG1), and levels of 3-nitrotyrosine (3-NT) were measured in muscle samples by Western immunoblotting. Whole-tissue extracts were prepared as described elsewhere [24]. Briefly, 50 µg proteins were separated on 12%–15% polyacrylamide gels (Bio-Rad Laboratories, Hercules, CA, USA), transferred onto polyvinylidene difluoride membranes (Bio-Rad Laboratories), and blocked for 1 h in 5% milk in Tris-buffered saline Tween (Bio-Rad Laboratories). Blots were probed with commercially available primary antibodies for OGG1 (1:2500, Abcam, Cambridge, MA, USA; #ab63942), TFR1 (1:1000, Cell Signaling Technology, Beverly, MA, USA; #13113), ZIP14 (1:1000, Sigma–Aldrich, St. Louis, MO, USA; #HPA016508), and 3-NT (1:1000, Cell Signaling Technology; #9691S). A custom-made polyclonal rabbit primary antibody was used for detecting ZIP8 (1:1000). The antibody was raised to a peptide [(NH_2_) FGNDNFGPQEKT (COOH)] selected from the full-length sequence [33] designed by Dr. Tolunay Beker Aydemir (University of Florida, Gainesville, FL, USA) who also performed the purification [34]. To allow affinity purification, a cysteine residue was added to the N terminus for coupling to the carrier protein and for conjugation to Sulfolink (Pierce, Rockford, IL, USA). The antibody was prepared in rabbit as previously described [35]. Anti-rabbit secondary antibody conjugated with horseradish peroxidase (1:10000, Cell Signaling Technology; #7074) was used to enable subsequent protein detection. Protein bands were visualised with SuperSignal West Femto Maximum Sensitivity Substrate (Thermo Scientific) using a ChemiDoc XRS imager (Bio-Rad Laboratories). Spot density of the target bands was normalised to the amount of protein loaded in each lane, as determined by densitometric analysis of the corresponding Ponceau S-stained membranes [36]. Bands were quantified using Image Lab 6.0 software (Bio-Rad Laboratories) according to the “Total Lane Protein” setting.

### 2.7. Quantification of Mitochondrial DNA (mtDNA) Content

Genomic DNA was purified from muscle samples using a Wizard Genomic DNA Purification Kit according to the manufacturer’s instructions (Promega, Madison, WI, USA). Briefly, 10–20 mg of muscle tissue were homogenised in 1 mL of nuclei cell lysis solution using a hard tissue disposable probe (Omni international, Kennesaw, GA, USA) on a PowerGen 500 homogenator (Thermo Fisher Scientific). Total DNA quantification was carried out on a NanoDrop 1000 spectrophotometer (Thermo Fisher Scientific) and integrity was verified by gel electrophoresis on 0.8% agarose gel in 1× Tris-borate-EDTA (TBE) (90 mM Tris-borate pH 7.4, 90 mM boric acid, 2.5 mM EDTA). Determination of mtDNA content was performed with the Human Mitochondrial DNA Monitoring Primer Kit (Takara Bio, Mountain View, CA, USA) using real-time polymerase chain reaction (RT-PCR). Amplification reactions were run on a CFX96 Touch™ Real-Time PCR Detection System (Bio-Rad Laboratories). Primers included in the kit specifically amplified mitochondrial genes corresponding to mitochondrial NADH dehydrogenase subunit 1 and 5 (ND1, ND5) and nuclear genes corresponding to solute carrier organic anion transporter family, member 2b1 (SLCO2B1), and serpin family A member 1 (SERPINA1) [37]. Melting curve analysis, non-template control reactions, and gel electrophoresis of PCR products were used to check amplification specificity of each experiment. Each sample was analysed in triplicate in 20 µL final volume. The reaction mixture consisted of 1× Terra qPCR Direct SYBR Premix (Takara Bio), 0.2 µM forward and reverse primers, and 10 ng of genomic DNA template. Amplification proceeded for 40 cycles. Quantification of relative mtDNA content was accomplished according to the Pfaffl mathematical model [38]. Differences in threshold cycle values for the ND1/SLCO2B1 pair (ΔCt1 = Ct for SLCO2B1 – Ct for ND1) and the ND5/SERPINA1 pair (ΔCt2 = Ct for SERPINA1 – Ct for ND5) were calculated, and the average of 2^ΔCt^ for the values of ΔCt1 and ΔCt2 was used as a measure of relative mtDNA abundance.

### 2.8. Analysis of mtDNA Damage

Quantitative RT-PCR was used to assess mtDNA damage according to the method described by Furda et al. [39] with minor adjustments. Briefly, 225 ng of purified total DNA was digested with PvuII Restriction enzyme (New England Biolabs, Ipswich, UK). Fifteen ng of digested DNA were used to amplify a 8.9-kb mtDNA fragment (accession number: J01415; 5′ sense position: 5999; 5′ antisense position: 14841) [39] with a TaKaRa LA Taq^®^ DNA Polymerase with GC Buffer (Takara Bio) and a 221-bp mtDNA fragment (accession number: J01415; 5′ sense position: 14620; 5′ antisense position: 14841) [39] with a DreamTaq DNA Polymerase (Thermo Fisher Scientific). Amplification was carried out using a CFX96 Touch™ PCR Detection System (Bio-Rad Laboratories) as described by Furda et al. [39]. Each sample was analysed in triplicate in 20 µL final volume. The reaction mixture for the 8.9-kb mtDNA fragment consisted of 1× GC Buffer I, 2U TaKaRa LA Taq^®^ DNA Polymerase (Takara Bio), 0.2 mM dNTPs, and 0.4 µM forward and reverse primers. The reaction mixture for the 221-bp mtDNA fragment included 1× DreamTaq Buffer (Thermo Fisher Scientific), 0.2 mM dNTPs, and 0.4 µM forward and reverse primers. Prior to quantification, amplification products of the 8.9-kb and the 221-bp fragments were electrophoresed on 0.8% agarose and 1.5% agarose gels, respectively, to check for PCR product specificity. Amplicons were quantified by Pico-Green (Thermo Fisher Scientific) using a Synergy HT multidetection microplate reader (BioTek) with excitation and emission wavelengths at 485 and 530 nm, respectively. Data obtained from the 221-bp mtDNA fragment were used to normalise results of the 8.9-kb fragment amplification. The number of mtDNA lesions was calculated using the equation: D = [1 − 2^−(Δ8.9-kb − Δ221-bp)^] × 10,000 bp/8900 bp [40].

### 2.9. Statistical Analysis

The normal distribution of data was ascertained through the Kolmogorov–Smirnov test. Comparisons for normally distributed continuous variables were performed by one-way analysis of variance (ANOVA) followed by Tukey′s post-hoc test when applicable. The non-parametric tests Mann–Whitney U and Kruskal–Wallis H (with Dunns′ post-hoc test as appropriate) were applied to assess differences for non-normally distributed continuous data. Differences in categorical variables among groups were determined via χ^2^ statistics. Correlations between variables were explored via Pearson′s or Spearman′s tests as appropriate. All analyses were performed using the GraphPrism 5.03 software (GraphPad Software, Inc., San Diego, CA, USA), with statistical significance set at *p* < 0.05.

## 3. Results

### 3.1. Characteristics of Study Participants

A total of 34 volunteers were enrolled, 11 young (six men and five women; mean age: 24.7 ± 4.4 years) and 23 older persons (14 men and nine women; mean age: 77.5 ± 8.0 years). Participant characteristics according to age groups and physical performance categories are shown in Table 1. No differences were observed among groups for gender distribution, BMI, or number of disease conditions and medications. The two subgroups of older adults did not differ for age. As per the study design, HF participants showed higher SPPB scores than LF older adults (*p* = 0.0002).

### 3.2. Quantification of Total Iron and Selected Metal Transporters in Vastus Lateralis Muscle Biopsies

**Figure 1:**
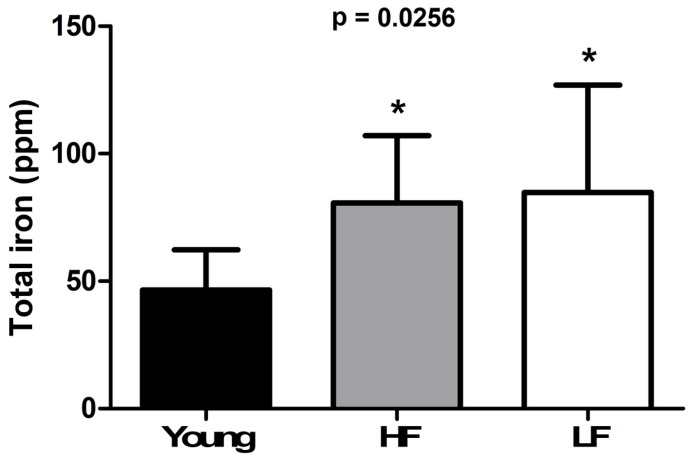
Total content of iron in the vastus lateralis muscle of young and old participants. Bars represent mean values (± standard deviation) in the three experimental groups. Values are expressed in ppm. * *p* < 0.05 vs. young group (*n* = 11). HF: high-functioning (*n* = 16); LF: low-functioning (*n* = 7).

To evaluate whether iron levels in muscle were associated with age and physical performance, total iron content was quantified by ICP-MS. Iron levels were significantly greater in muscles of old enrolees compared with the young group (*p* < 0.05), with no differences between SPPB categories (Figure 1).

**Figure 2:**
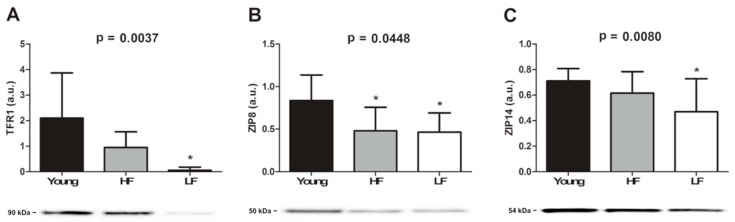
Protein expression of (**A**) transferrin receptor 1 (TFR1), (**B**) Zrt-Irt-like protein (ZIP) 8, and (**C**) ZIP14 in the vastus lateralis muscle of young and old participants. Bars represent mean values (±standard deviation) in the three experimental groups. Values are expressed in arbitrary units (a.u.). Representative blots are shown. * *p* < 0.05 vs. young group (*n* = 11). HF: high-functioning (*n* = 16); LF: low-functioning (*n* = 7).

Protein levels of selected iron transporters (TFR1, ZIP8, and ZIP14) were assayed by Western immunoblotting. The expression of TFR1, the primary cellular iron importer, was significantly lower in old LF participants compared with the young group (*p* < 0.05; Figure 2A). Also, lower protein levels of ZIP8 were detected in old enrolees compared with their younger counterparts (Figure 2B). A pattern similar to TFR1 was found for ZIP14 (*p* < 0.05; Figure 2C).

### 3.3. Circulating Levels of Ferritin, Hepcidin, and Selected Inflammatory Biomarkers

Perturbations in iron status have been associated with chronic low-grade inflammation during ageing [41]. In turn, inflammation is acknowledged as a major mechanism contributing to functional impairment [42]. We, therefore, verified whether circulating levels of ferritin, hepcidin, and selected inflammatory biomarkers were associated with age and functional status.

**Figure 3:**
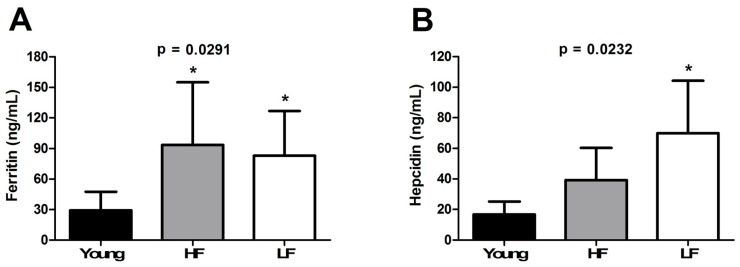
Plasma levels of (**A**) ferritin and (**B**) hepcidin in young and old participants. Bars represent mean values (± standard deviation) in the three experimental groups. * *p* < 0.05 vs. young group (*n* = 11). HF: high-functioning (*n* = 16); LF: low-functioning (*n* = 7).

An age-dependent increase was observed for plasma ferritin concentrations (*p* = 0.0291; Figure 3A), with no differences between SPPB categories. Circulating levels of the defensin-like hormone hepcidin were also increased with age (*p* = 0.0232; Figure 3B). The post-hoc test revealed significantly higher hepcidin concentrations in LF older adults compared with young enrolees.

**Figure 4:**
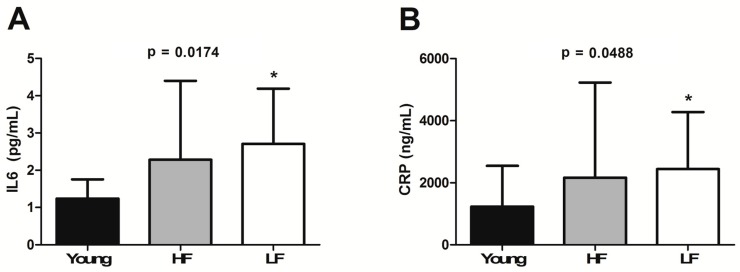
Plasma levels of (**A**) interleukin 6 (IL6) and (**B**) C-reactive protein (CRP) in young and old participants. Bars represent mean values (±standard deviation) in the three experimental groups. * *p* < 0.05 vs. young group (*n* = 11). HF: high-functioning (*n* = 16); LF: low-functioning (*n* = 7).

A similar pattern was described for plasma IL6 (*p* = 0.0174; Figure 4A) and CRP (*p* = 0.0488; Figure 4B).

We performed a correlation analysis to test the hypothesis of an association between inflammation and iron status in LF older adults. As reported in Table 2, ZIP14 was the only iron transporter showing a significant negative correlation with IL6.

### 3.4. Determination of mtDNA Content and Damage

**Figure 5:**
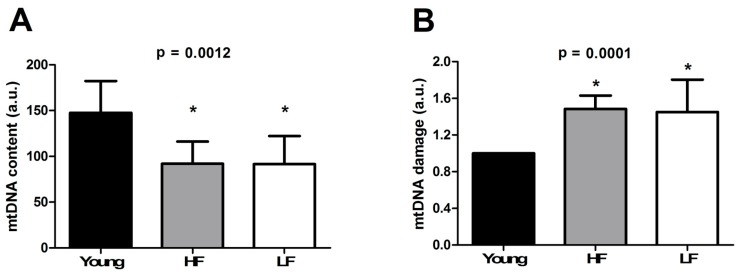
(**A**) mtDNA content and (**B**) mtDNA damage in the vastus lateralis muscle of young and old participants. Bars represent mean values (± standard deviation) in the three experimental groups. * *p* < 0.05 vs. young group (*n* = 11). HF: high-functioning (*n* = 16); LF: low-functioning (*n* = 7).

mtDNA homeostasis in muscle becomes impaired during ageing and in the setting of atrophying conditions [43]. However, whether the abundance and integrity of mtDNA in muscle are associated with physical function in old age is still debated [44]. In the attempt to shed light on this relevant research question, we determined the relative content of mtDNA and mtDNA damage load in muscle samples of young and old enrollees. As depicted in Figure 5, older participants showed lower mtDNA content (*p* = 0.0012, Figure 5A) and greater mtDNA damage (*p* = 0.0001, Figure 5B) compared with young controls, with no differences between HF and LF individuals.

### 3.5. Protein Levels of Selected Markers of Oxidative/Nitrosative Damage

Protein expression of the repair enzyme OGG1 and levels of nitrosative stress-associated 3-NT were determined in muscle samples to obtain indications on the extent of oxidative-related molecular damage [45].

**Figure 6:**
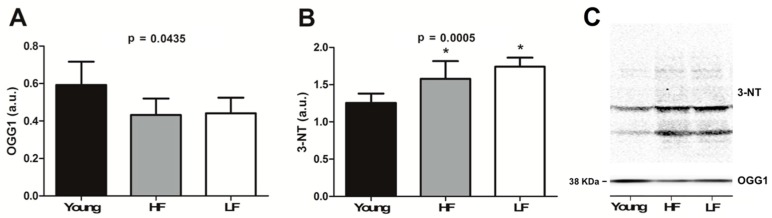
Protein expression of (**A**) 8-oxoguanine DNA glycosylase (OGG1) and (**B**) levels of 3-nitrotyrosine (3-NT) in the vastus lateralis muscle of young and old participants. Bars represent mean values (±standard deviation) in the three experimental groups. Representative blots of OGG1 and 3-NT are shown in panel (**C**) a.u. arbitrary units. * *p* < 0.05 vs. young group (*n* = 11). HF: high functioning (*n* = 16); LF: low functioning (*n* = 7).

OGG1 protein expression declined with ageing (*p* = 0.0435), with no differences among individual groups (Figure 6A). An age-related increase in 3-NT levels was observed (*p* = 0.0005, Figure 6B), without differences between the two old groups.

## 4. Discussion

Iron homeostasis is altered in muscle of old rodents, possibly contributing to muscle fibre atrophy and loss via oxidative stress-mediated signalling pathways [18]. A specific form of non-apoptotic cell death, referred to as ferroptosis, seems to occur upon intracellular iron overload, causing oxidative injury which probably involves lipid peroxidation [46]. This iron-driven cell death may operate via mitochondrial and NADPH-dependent oxidases reactive oxygen species burst [46]. However, to the best of our knowledge, the relationship between iron status and physical function in old people was not previously explored.

Studies from our group showed increased levels of muscle non-haem iron, including labile fraction, with age in old rats following hind limb suspension [18]. Such changes were associated with elevated expression of ferritin and decreased TFR1 content [18]. Age-dependent iron accumulation was also reported in muscle subsarcolemmal mitochondria of rats [47]. Notably, mitochondrial iron levels were shown to impact organelle RNA damage as well as the susceptibility to opening of the mitochondrial permeability transition pore [47]. This prompted us to test the hypothesis of a relationship between iron status and age-related functional decline involving muscular mitochondrial damage.

Our finding of an age-dependent accumulation of iron in skeletal muscle (Figure 1) paralleled by decreased expression of two of the three metal importers assayed (i.e., TFR1 and ZIP14) in the LF group (Figure 2A–C) supports the idea of a link between iron dyshomeostasis in muscle and functional status. The analysis of iron-related circulating factors offered further insights into this association. Indeed, ferritin levels, an indicator of stored iron, were found to be higher in both HF and LF older adults (Figure 3A), which might arise from chronic inflammation [48]. This view is consistent with our observation of an age-dependent elevation of plasma IL6 and CRP, the levels of which were both higher in LF relative to HF participants (Figure 4A,B).

Although in apparent contrast to our original hypothesis, the measurement of circulating levels of hepcidin provided interesting information regarding such an association. This defensin-like hormone, produced mainly by the liver, plays a major role in modulating iron metabolism [11]. Indeed, via binding to the iron exporter ferroportin at the level of key iron sources [i.e., duodenal enterocytes (absorption of dietary iron), splenic and hepatic macrophages (recycling iron from erythrophagocytosis), and hepatocytes (iron stores)], hepcidin induces its own endocytosis and lysosomal degradation as well as of ferroportin [12]. As a consequence, decreased intestinal absorption and release of iron from recycling macrophages occur, ultimately resulting in lower circulating iron concentrations [11].

In the present investigation, higher levels of hepcidin were found in older participants, especially in those classified as LF (Figure 3B), with a parallel increase in IL6 and CRP (Figure 4A,B). These results are in line with previous reports pointing to IL6 as a major hepcidin inducer in older adults [49,50,51], in whom it may be responsible for iron-limited erythropoiesis [52,53]. Whether inflammation reduces iron availability for myoglobin assembly, thereby contributing to impairing muscle function, is presently unknown. Further support to the link among inflammation, iron status, and functional impairment is lent by the strong negative correlation (*r* = −0.99, *p* = 0.04) between circulating IL6 levels and muscle expression of ZIP14 in LF older participants (Table 2). Although our experimental design does not allow inferring about a direct involvement of ZIP14 in muscle iron clearance, a link between ZIP14 expression and IL6 induction has previously been reported and a role for ZIP14 in iron uptake has been hypothesised [54].

A hepcidin-independent regulation of iron status with ageing cannot be excluded. Indeed, studies conducted in older adults with anaemia and chronic inflammation did not detect increased levels of hepcidin in urine or serum [55,56]. In this context, the co-occurrence of multiple age-related conditions may explain changes in the iron status [57,58]. This could be the case for higher circulating ferritin levels in HF participants, which may result, for instance, from the stimulation of ferritin expression by reactive species [59,60].

mtDNA content and damage (Figure 5A,B) as well as the expression of OGG1, one major enzymatic system of mtDNA base excision repair, and 3-NT (Figure 6A,B) showed an age-related association rather than changes dependent on functional status. These findings are in line with previous results in other aged post-mitotic tissues [61].

Taken as a whole, results from the present study suggest that altered iron metabolism during ageing may predispose to oxidant generation and damage to cell components, including mitochondria. In particular, the association of iron dyshomeostasis with systemic inflammation might represent a kingmaker towards functional decline. Disruption of iron metabolism in myocytes might therefore represent a novel target for interventions aimed at preserving muscle health in old age.

## 5. Limitations of the Study

While reporting novel findings, our work is not devoid of limitations that need to be discussed. First of all, the study is exploratory in nature due to the small sample size and the limited amount of muscle tissue available for analyses. In addition, the cross-sectional design hampers inference about the time course of changes in analysed mediators and the development of functional decline. Also, only total iron levels were measured and no information is available about haem and non-haem iron. Likewise, neither haemoglobin levels nor mean corpuscular haemoglobin concentration in erythrocytes were measured. Furthermore, plasma iron levels, Tf affinity and saturation, and ferritin capacity were not assessed, which impeded a comprehensive appraisal of body iron homeostasis. Finally, the study did not include a group of actively exercising older people. Both categories of old participants were physically inactive and this did not allow appreciating the possible effect of physical activity on iron status in muscle in old age.

## Figures and Tables

**Table 1:** Participant characteristics according to age groups and physical performance categories.

		Old (*n* = 23)		
Characteristic	Young (*n* = 11)	HF (*n* = 16)	LF (*n* = 7)	*p* Value
Age (years), mean ± SD	24.7 ± 4.4	76.0 ± 6.0 *	81.0 ± 3.7 *	<0.0001
Gender (female), n (%)	5 (45.5)	4 (25.0)	5 (71.4)	0.1076
BMI (kg/m^2^), mean ± SD	24.9 ± 4.2	27.7 ± 3.6	27.8 ± 4.2	0.1604
Number of diseases ^¥^, mean ± SD	1.0 ± 0.8	1.9 ± 1.4	2.1 ± 1.8	0.1274
Number of medications ^#^, mean ± SD	2.9 ± 2.6	3.7 ± 3.2	1.7 ± 1.4	0.3112
SPPB summary score, mean ± SD	--	11.4 ± 0.5	6.1 ± 1.7 ^§^	0.0002

Abbreviations: BMI, body mass index; HF, high functioning; LF, low functioning; SD, standard deviation; SPPB, Short Physical Performance Battery. * *p* < 0.05 vs. young group. ^§^ *p* < 0.05 vs. HF. ^¥^ includes hypertension, coronary artery disease, prior stroke, peripheral vascular disease, diabetes, chronic obstructive pulmonary disease, and osteoarthritis. ^#^ includes prescription and over-the-counter drugs.

**Table 2:** Relationship between plasma concentrations of IL6 and protein expression of iron transporters in muscle in old low-functioning participants.

	TFR1	ZIP14	ZIP8
Pearson r	−0.09161	−0.9976	0.5968
95% confidence interval	−0.8408–0.7779	0.0444	−0.4167–0.9488
R square	0.008392	0.9951	0.3561
p value (two-tailed)	0.863	0.0444	0.2111

Abbreviations: TFR1, transferrin receptor 1, ZIP, Zrt-Irt-like protein.
